# Influence of Root Exudates on the Bacterial Degradation of Chlorobenzoic Acids

**DOI:** 10.1155/2013/872026

**Published:** 2013-10-10

**Authors:** Blanka Vrchotová, Petra Lovecká, Milena Dražková, Martina Macková, Tomas Macek

**Affiliations:** Department of Biochemistry and Microbiology, Faculty of Food and Biochemical Technology, Institute of Chemical Technology Prague, Technicka 3, 166 28 Prague 6, Czech Republic

## Abstract

Degradation of chlorobenzoic acids (e.g., products of microbial degradation of PCB) by strains of microorganisms isolated from PCB contaminated soils was assessed. From seven bulk-soil isolates two strains unique in ability to degrade a wider range of chlorobenzoic acids than others were selected, individually and even in a complex mixture of 11 different chlorobenzoic acids. Such a feature is lacking in most tested degraders. To investigate the influence of vegetation on chlorobenzoic acids degraders, root exudates of two plant species known for supporting PCB degradation in soil were tested. While with individual chlorobenzoic acids the presence of plant exudates leads to a decrease of degradation yield, in case of a mixture of chlorobenzoic acids either a change in bacterial degradation specificity, associated with 3- and 4-chlorobenzoic acid, or an extension of the spectrum of degraded chlorobenzoic acids was observed.

## 1. Introduction

There are many different known and used methods for decontamination of polluted areas [1]. It is possible to use biological or physical or chemical methods or combination of them. Each of these approaches has its positive but also negative aspects (Table 1). In this paper use of biological method, microorganisms, and plant, for removal of chlorobenzoic acids (CBAs) is discussed.

CBAs are known as degradation products generated during the bacterial degradation of substances such as polychlorinated biphenyls (PCBs), DDT, and other chlorinated xenobiotics. Microbial degradation of such compounds can result in the formation of CBAs with different numbers and positions of chlorine atoms on an aromatic ring [2]. Degradation of CBAs and its parent compounds (e.g., PCBs) is usually performed by at least two different bacterial strains. 

Microbial degradation of CBAs, as in the degradation of other chlorinated aromatic compounds, is confounded by the presence of chlorine atoms which interfere with the enzymatic breakdown of the aromatic ring. This problem can be overcome by the incorporation of a dehalogenation step at the beginning of the catabolic pathway as in the 4-CBA degradation pathway [3]. The first step of 4-CBA degradation is the conversion to 4-hydroxybenzoate by a hydrolytic process catalyzed by 4-chlorobenzoate dehalogenase with acetyl coenzyme A [3]. The hydrolytic process is followed by breakdown of the aromatic ring.

2-CBA can be degraded by two different degradation pathways. The most common is the spontaneous removal of chlorine and CO_2_ from 2-CBA during the 1,2-dioxygenase attack resulting in the formation of catechol. The second route is 1,6-dioxygenase attack resulting in the formation of 3-chlorocatechol [4]. The formation of 3-chlorocatechol is followed by cleavage of the aromatic ring resulting in formation of chloromuconic acid followed by a dechlorination step [4].

Degradation of 3-CBA can be processed through a number of different degradation pathways. Formation of the 3-chlorocatechol or 4-chlorocatechol is catalyzed by the chlorobenzoate-1,2-dioxygenase [5]. Protocatechuate (3,4-dihydroxybenzoic acid) or 5-chloroprotocatechuate (5-chloro-3,4-dihydroxybenzoic acid) can be formed by the 3-chlorobenzoate-3,4-dioxygenase [6]. Another possibility of the 3-CBA degradation is the formation of gentisate (2,5-dihydroxybenzoic acid) with 3-hydroxybenzoic acid as an intermediate [7]. 

In the case of dichlorinated CBAs, degradation proceeds mostly by a 1,2-dioxygenase attack [8]. Another option for degradation of dichlorinated CBAs includes the complete removal of chlorine atoms via a two-step process prior to aromatic ring cleavage like in 2,4-CBA degradation or a 3,4-dioxygenase attack like in 3,4-CBA degradation. The two steps of chlorine removal in 2,4-CBA degradation are reductive dechlorination leading to the formation of 4-CBA and hydrolytic dechlorination. The degradation of 3,4-CBA is initiated by a 3,4-dioxygenase attack resulting in the formation of 5-chloroprotocatechuate [4].

As described, there is a wide variety of CBA degradation pathways which leads to the possibility that different CBAs can be degraded by one bacterial strain with enzymes from more than one route. These enzymatic pathways may potentially be induced by plant secondary compounds, root exudates, or the other CBAs presented [9].

This precondition was investigated by testing microbial CBAs degradation abilities of strains previously isolated from PCB contaminated soils. Three bacterial strains were isolated from soil collected at the paint company COLORLAK in Uherské Hradiště (UH strains) [10] and four strains from Žamberk company ZEZ SILKO (A strains) [11]. These experiments test the degradation activity of strains by growth on multiple different individual CBAs. This degradation potential was then compared with the degradation potential determined in the set of tests with a mixture of all eleven tested CBAs. In addition to the testing of degradation abilities, the impact of plant exudates, prepared by hydroponic cultivation of tobacco (*Nicotiana tabacum*) or black nightshade (*Solanum nigrum*) plants, on degradation of individual CBAs or a mixture of eleven CBAs was determined.

## 2. Materials and Methods

### 2.1. Chemicals

Chlorobenzoic acids (2-CBA; 3-CBA; 4-CBA; 2,3-CBA; 2,4-CBA; 2,5-CBA; 2,6-CBA; 3,4-CBA; 3,5-CBA; 2,3,5-CBA, and 2,4,6-CBA) were obtained from Sigma-Aldrich. All chemicals used in the cultivation media were of analytical reagent grade. Mobil phases used for liquid chromatography were of HPLC gradient grade. 

### 2.2. Bacterial Strains

Bacterial strains were previously isolated from PCBs contaminated soil from two different locations in the Czech Republic. Strains A7, A8, A18, and A19 were obtained from Žamberk soil contaminated with a PCB mixture Delor 103 of 300 mg per kg dry soil [11]. Strains UH82, UH133, and UH222 were from Uherské Hradiště soil containing 0–470 mg of total PCBs per gram dry weight of soil [10]. Strains A7 and A8 were identified as *Pseudomonas fluorescens*, strain A18 as *Pseudomonas pseudoalcaligenes, *and strain A19 as *Pseudomonas stutzeri *[11]. Strains UH133 and UH222 from Uherské Hradiště were classified as *Pandoraea* sp.; strain UH82 was identified as *Arthrobacter *sp. [10].

### 2.3. Preparation of Plant Root Exudates

Plant root exudates were prepared from both plant species according to the same procedure. One-month-old plants grown in soil were washed with water and with sterile tenth-strength minimal media (3.5 g L^−1^ K_2_HPO_4_; 1.5 g L^−1^ KH_2_PO_4_; 0.5 g L^−1^ NH_4_Cl; 0.5 g L^−1^ NaCl; 0.14 g L^−1^ Na_2_SO_4_; 0.07 g L^−1^ MgCl_2_). Stem of the plant was wrapped with gaze stopper above the roots and placed into a 250 mL Erlenmeyer flask with 200 mL of tenth-strength minimal medium. As shade aluminum foil covered the bottom and sides of the flask to the level of the medium. These prepared plants were hydroponically cultivated in 16-hour day light in 24°C for one month. Losses by evaporation were refilled with distilled water. Collected solutions containing root exudates were combined, and mineral salts were added to the solution to obtain full minimal medium with plant root exudates. Medium was filter-sterilized and stored at 4°C. This way of root exudates preparation was chosen to obtain plant exudates profile most similar to soil-grown plants.

### 2.4. Cultivation Experiments with Different CBAs

Bacterial strains were cultivated in liquid minimal medium with the addition of 200 mg L^−1^ of benzoic acid on a rotary shaker (130 rpm) at 28°C. After two days cells were harvested by centrifugation (10 min 5000 ×g) and washed twice in minimal medium. Degradation of CBA was measured in 8 mL glass vials. The total volume of the cultivation mixture was 2 mL. The mixture consisted of either single CBAs (200 mg L^−1^) or a mixture of 11 CBAs (20 mg L^−1^ of each CBA), bacteria (OD_600_ = 0.4), and either minimal medium or minimal medium with root exudates from tobacco or black nightshade. CBA concentrations were measured by HPLC at time zero and after 5 days of bacterial cultivation. Sample of medium was centrifuged before measurement on HPLC (6 min. × 10 000 g) and stored at −18°C. CBA degradation was expressed as the difference between the initial concentration and the concentration of CBA in the medium after 5 days of cultivation. As a control, appropriate medium with the addition of CBA without bacteria was used. For the removal of outliers the Dean Dixon test (*α* = 0.05) was used. Degradation was evaluated by comparison with control by one way ANOVA (with 90% significance).

### 2.5. HPLC Analysis of CBA Concentration

The concentration of added CBAs was quantified by reverse phase high pressure liquid chromatography (HPLC) (Hewlett Packard model 1100) computer operated system with Chemstation program. HPLC analytical procedures employed column LUNA C18(2) 150 mm × 2.00 mm × 5 *μ*m (Phenomenex, USA). Isocratic elution with mobile phase methanol (water 1% (*v/v*) H_3_PO_4_ 60 : 40 for 2,3,5-CBA and 2,4,6-CBA and 55 : 45 for other CBAs) was used. Mobile phase flow rate was 0.4 mL min^−1^, injection volume 10 *μ*L, and the detection on DAD detector at the wavelength 205 nm. For HPLC measurement of CBA mixture, column Kinetex C18 100A 150 mm × 2.10 mm × 2.6 *μ*m (Phenomenex, USA) was used with mobile phase that consisted of 45 : 55 methanol : buffer pH 3.18 (41.8 mmol L^−1^ H_3_PO_4_; 55.8 mmol L^−1^ H_3_BO_3_; 50.0 mmol L^−1^ NaOH; 55.1 mmol L^−1^ CH_3_COOH) and flow rate 0.15 mL min^−1^. All other parameters were the same as those previously described.

## 3. Results and Discussion

### 3.1. Degradation of Single CBA

**Figure 1:**
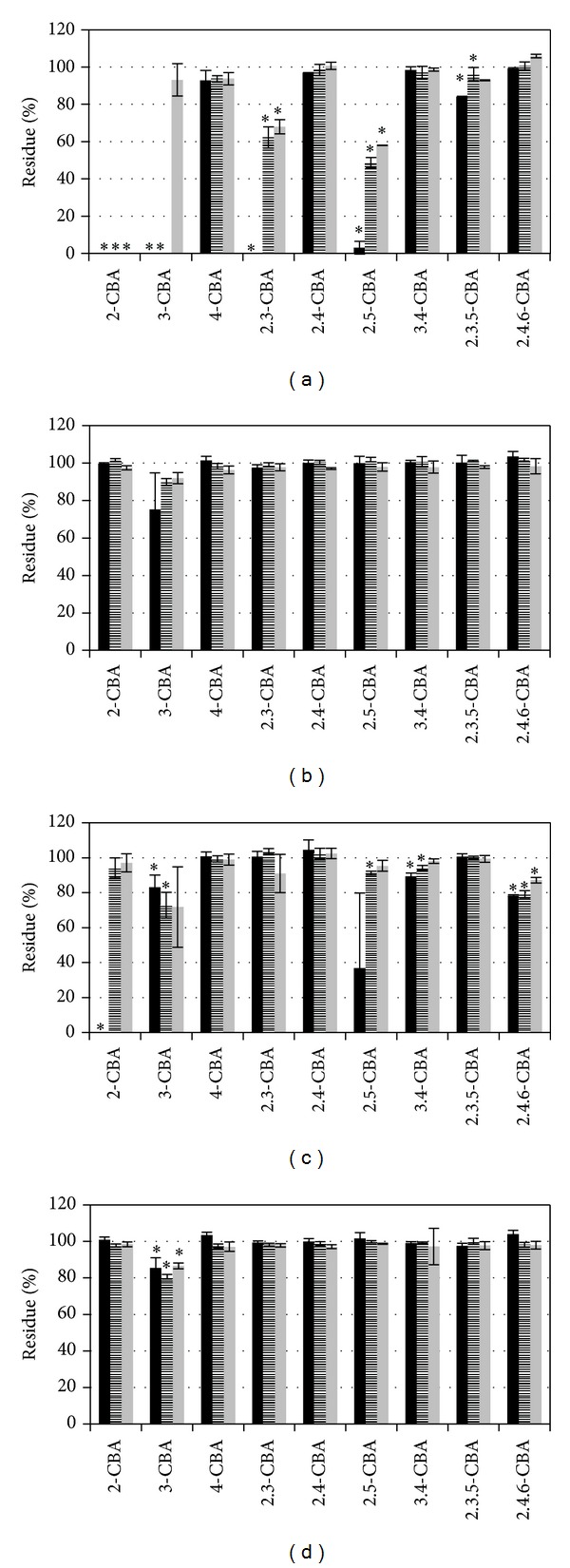
Degradation of single CBA ((a), (c)) and mixture of CBA ((b), (d)) by strains A7 (*Pseudomonas fluorescens*) ((a), (b)) and A8 (*Pseudomonas fluorescens*) ((c), (d)). Black bar in minimal medium, stripped bar in minimal medium with exudates of black nightshade (*Solanum nigrum*), and grey bar in minimal medium with exudates of tobacco (*Nicotiana tabacum*). Stars indicate the statistically significant CBA removal (ANOVA).

**Figure 2:**
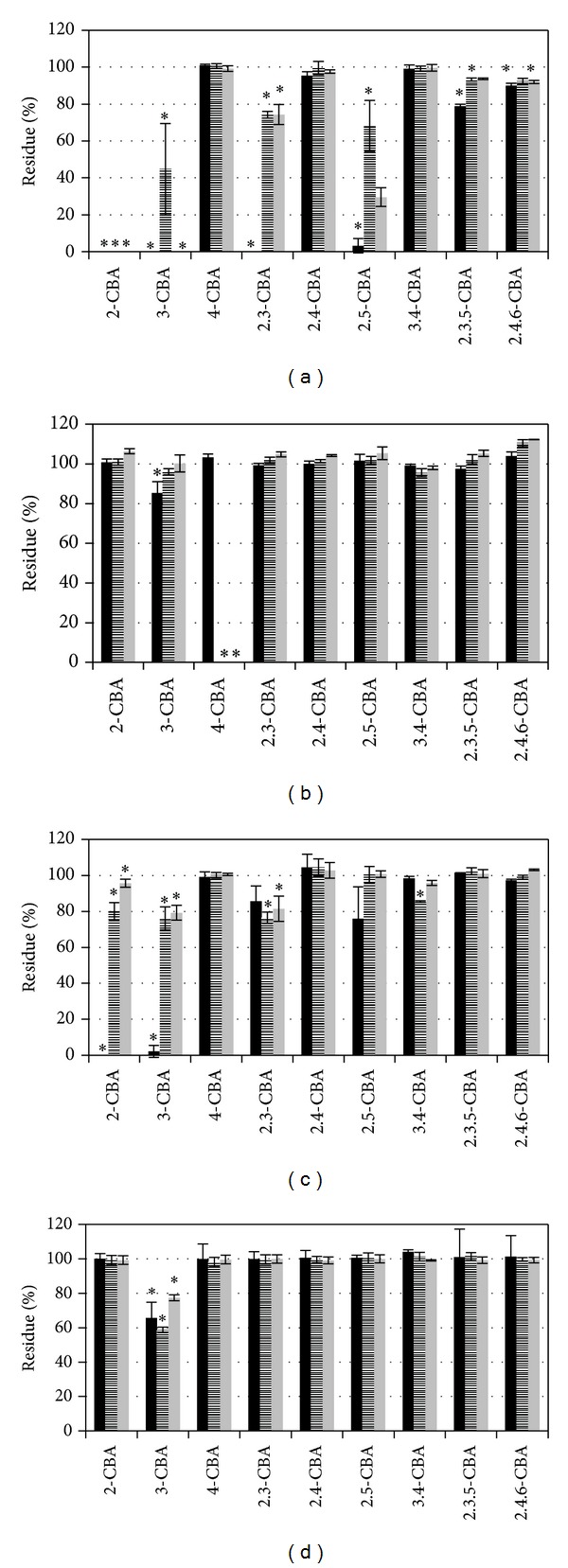
Degradation of single CBA ((a), (c)) and mixture of CBA ((b), (d)) by strains A18 (*Pseudomonas pseudoalcaligenes*) ((a), (b)) and A19 (*Pseudomonas stutzeri*) ((c), (d)), Black bar in minimal medium, stripped bar in minimal medium with exudates of black nightshade (*Solanum nigrum*), and grey bar in minimal medium with exudates of tobacco (*Nicotiana tabacum*). Stars indicate the statistically significant CBA removal (ANOVA).

Strains from Žamberk soil were able to degrade a greater variety of CBAs than strains from Uherské Hradiště when CBAs were supplied individually. Strains from Žamberk can be divided into two groups with similar degradation profiles: the first group containing strains A7 and A18 and the second consists of strains A8 and A19 (Figures 1 and 2). 

**Figure 3:**
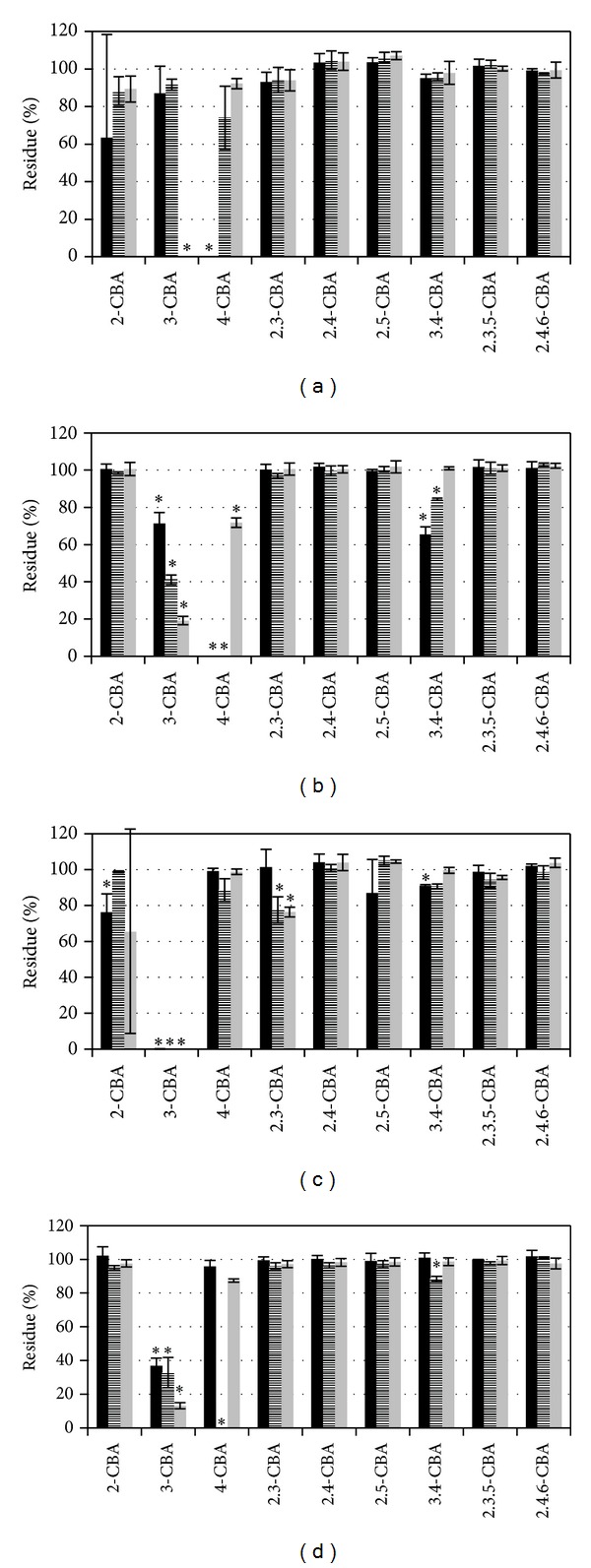
Degradation of single CBA ((a), (c)) and mixture of CBA ((b), (d)) by strains UH82 (*Arthrobacter *sp.) ((a), (b)) and UH133 (*Pandoraea *sp.) ((c), (d)). Black bar in minimal medium, stripped bar in minimal medium with exudates of black nightshade (*Solanum nigrum*), and grey bar in minimal medium with exudates of tobacco (*Nicotiana tabacum*). Stars indicate the statistically significant CBA removal (ANOVA).

**Figure 4:**
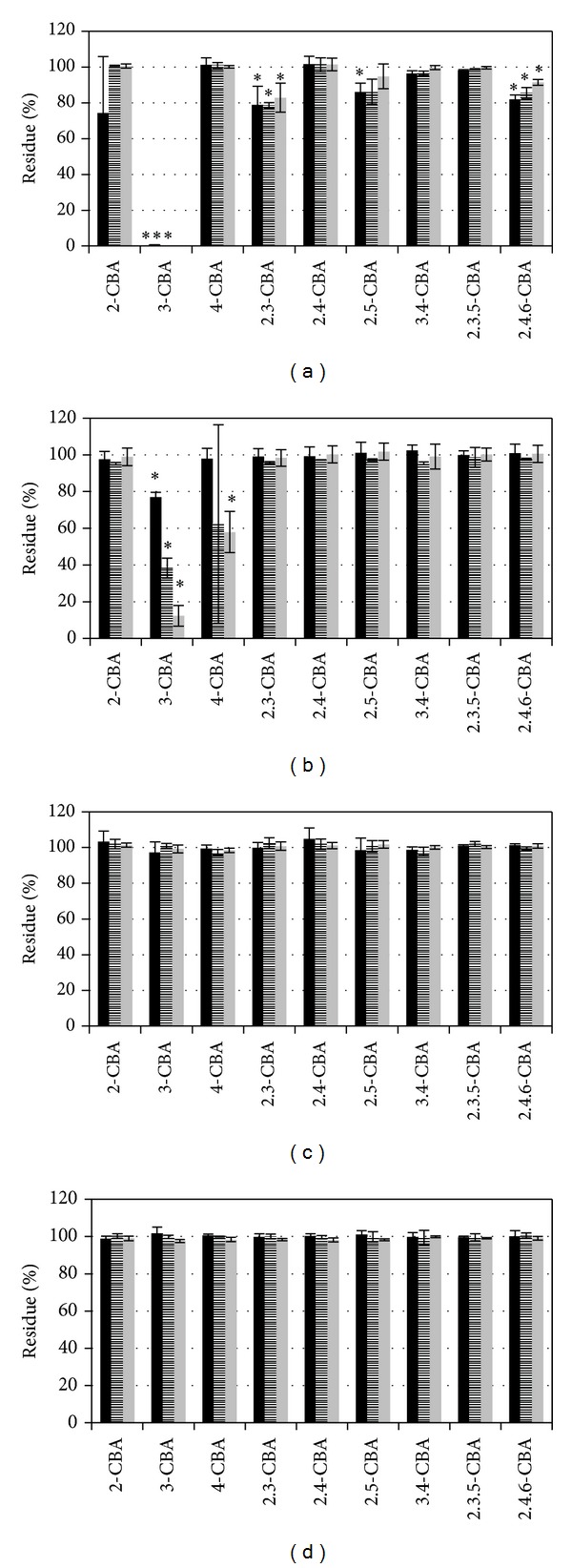
Degradation of single CBA ((a), (c)) and mixture of CBA ((b), (d)) by strains UH222 (*Pandoraea *sp.) ((a), (b)) and control ((c), (d)). Black bar in minimal medium, stripped bar in minimal medium with exudates of black nightshade (*Solanum nigrum*), and grey bar in minimal medium with exudates of tobacco (*Nicotiana tabacum*). Stars indicate the statistically significant CBA removal (ANOVA).

Most of the strains from Uherské Hradiště degraded only 3-CBA except strains UH82 and UH222 (Figures 3 and 4). Strain UH82 degraded only 4-CBA (Figure 3). Strain UH222 in addition to the 3-CBA partially degraded also 2,3-CBA and 2,4,6-CBA. Degradation of 2,6-CBA and 3,5-CBA is not shown in pictures because of no degradation in all tested possibilities.

Earlier research has focused on the abilities of different bacterial strains to degrade various CBAs individually added [4] or in a combination of CBAs that consisted of no more than three CBAs [12, 13]. These tests showed that some microbial strains can grow on a wide range of monohalogenated and dihalogenated CBAs [4, 14, 15]. Several strains can also use trihalogenated CBAs as the sole carbon source [4, 12, 16].

Tested strains proved a high heterogeneity of CBAs degradation ability when CBAs were supplied individually and served as a sole source of carbon and energy. Under these conditions a slight degradation activity of trichlorinated CBAs was also registered. Strains A7 and A18 degraded 2,3,5-CBA, and strains A8, A18 and UH222 degraded 2,4,6-CBA. 

Based on the results it can be concluded that degradation of CBAs with chlorine in -*ortho* position like 2-CBA, 2,3-CBA, and 2,5-CBA was very common and degradation of 3-CBA was more frequent then degradation of 4-CBA. On the contrary degradation of CBAs with chlorine atoms in both -*ortho* positions (2,6-CBA or 2,4,6-CBA) was very rare, the same as degradation of CBAs with chlorines in both -*meta* positions (3,5-CBA and 2,3,5-CBA). 

### 3.2. Degradation of CBA Mixture

The second part of this work was a comparison of the degradation potential assessed in individual CBA degradation tests with results obtained by testing bacterial growth on a mixture of eleven CBAs. To the authors knowledge, this type of measurement was never done previously for bacteria except in a study assessing the degradation potential of ligninolytic fungi [17]. 

When CBAs are present in a mixture, some of them can cause inhibition of degradation of other CBAs present. The presence of 3,5-CBA completely inhibited growth of *Pseudomonas putida* P111 on 2-CBA, 2,3-CBA, 2,4-CBA, 2,5-CBA, and 2,3,5-CBA [12]. Also, the presence of 2,6-CBA slowed down 4-CBA degradation, while 2,3-CBA and 3,4-CBA completely inhibited the degradation of 4-CBA [18]. 

Effect of certain CBAs on the degradation of other CBAs is difficult to determine in this study because all eleven CBAs were tested together. Degradation tests with the mixture of eleven CBAs showed that the number of degraded CBAs was mostly reduced in comparison with the number of degraded CBAs in tests where CBAs were added individually. The most sensitive strains (from the point of the decrease of degradation) were strains A7 and A8 (Figure 1).

On the contrary, due to the presence of other CBAs, in the mixture can be same CBA degraded co-metabolically [16]. Induction effects on CBA degradation can be seen in strain UH82. In individual CBA degradation tests, strain UH82 degraded only 4-CBA; however, in degradation test with the mixture of CBAs the strain UH82 degraded 3-CBA and 3,4-CBA in addition to 4-CBA (Figure 3). 

From the results of mixture degradation it is possible to conclude that in tested strains degradation ability of 3-CBA is most stable. In individual tests all strains except UH82 proved 3-CBA degradation, and in mixture CBAs degradation, five strains preserved 3-CBA degradation ability.

### 3.3. Impact of Plant Root Exudates on CBA Degradation Potential

Microbial degradation potential of xenobiotics can be affected by abiotic factors (composition of pollutants), as well as by biotic factors, including the influence of other organisms present in the environment. The presence of plants or plant secondary metabolites may affect the degradation of CBAs [19, 20]. The plants themselves have the CBAs degradation potential [21, 22]. Plants of *Salix viminalis* were able to remove at least 20% of 4-CBA from cultivation medium [22]. Plant cell cultures of black nightshade and horseradish metabolized in 2 weeks more than 90% of 2-CBA, 20–40% of 2,3-CBA, 2,4-CBA, 2,5-CBA, and 2,6-CBA at concentration of 50 mg/L [21].

The presence of plants can also increase CBA degradation by induction of microbial degradation [13, 23] or excretion of plant root exudates [24]. Plant exudates from *Elymus dauricus *have been shown to reduce 2-CBA levels [13]. Hernandez et al. [25] demonstrated that the amendment of soil by plant residues containing terpenes can promote the metabolism of PCBs. 

In experiments where we tested root exudates from black nightshade and tobacco, changes were observed in degradation specificity caused by the addition of plant root exudates. For most strains, plant root exudates stopped or slowed degradation of CBAs when CBAs were added either individually or in a mixture (Figures 1–4). However, there were three strains which did not follow this pattern when exposed to 3-CBA and 4-CBA. Strain A18 originally degraded 2-CBA and 3-CBA (Figure 2) when CBAs were tested individually in the presence of exudates from both tested plant species. The same A18 strain, with the mixture of CBAs, acquired 4-CBA degradation activity. Strain UH82 lost the potential to degrade 4-CBA but obtained 3-CBA degradation activity when exudates of tobacco were used for individual or CBA mixture degradation tests (Figure 3). Exudates of black nightshade did not have this effect on strain UH82 in individual CBA degradation tests, but black nightshade exudates preserved 4-CBA degradation activity and induced 3-CBA degradation in the mixture of CBAs. Strain UH133, in the presence of black nightshade exudates, obtained 4-CBA degradation activity on CBAs mixture (Figure 3); however, tobacco exudates did not induce 4-CBA activity.

These changes in specificity can be generated due to induction or inhibition of enzymes from different CBA pathways by compounds present in plant root exudates. Most of the plant exudates contain 4-hydroxybenzoic acid [26], an intermediate in the 4-CBA degradation pathway. The presence of 4-hydroxybenzoic acid can either inhibit the degradation of 4-CBA (inhibition of reaction by high concentration of product), as in the case of strain UH82 with tobacco root exudates, or induce the degradation of 4-CBA as in strain UH133 cultivated with black nightshade root exudates. In the second case the induction can be caused by stimulation of 4-hydroxybenzoic acid degradation pathway which can consequently lead to stimulation of 4-CBA degradation pathway. In the case of strain UH133 this effect could be so dominant that it could cause a preference of 4-CBA degradation prior to 3-CBA degradation. 

## 4. Conclusion

This study clearly demonstrated that from the strain degradation ability determined on individually added CBA it is difficult to define strain degradation effectiveness on a complex mixture of CBAs. Therefore, practical use of microbial strains for decontamination requires choosing strains with wide degradation abilities determined on mixture of pollutants. From strains that were tested, most promising ones are A18 and UH82, thanks to their degradation of CBAs mixture. Also cooperation of different organisms can increase efficiency of decontamination, for example, cooperation of plants and microorganisms. Results indicate that combination with black nightshade will be potentially most suitable for decontamination.

## Figures and Tables

**Table 1:** Comparison of technologies used for decontamination of polluted areas [1].

Technology	Description	+	−
Bioremediation	Use of plants and/or microorganisms	(i) *In situ*/*ex situ* (ii) Cheap (iii) Economical also for low level contamination	(i) Needs longer time (ii) Contaminant specific

Physical methods			
Burning/dump site	Excavation of the contaminated soil and its burning at high temperature or deposition on a dump side	(i) *Ex situ* (ii) Fast (iii) Possible to use for wide spectra of contaminants	(i) Destruction of environment (ii) Needs special equipment (iii) Burning effective just for organic contaminants
Solidification/stabilization	Immobilization of contaminant on sorbent	(i) *In situ*/*ex situ* (ii) Fast	(i) Possible rebounding contaminant is present forever
Electrokinetic remediation	Removal of contaminants by electromigration and electroosmosis	(i) *In situ*/*ex situ* (ii) Fast (iii) Organic and heavy metals	(i) Needs special equipment (ii) Not possible to use for all types of soils
Washing/flushing	Use of water or detergent solution for washing of contaminants	(i) *In situ*/*ex situ* (ii) Possible to use for wide spectra of contaminants	(i) Needs special equipment (ii) Need of decontamination of the resulting solution

Chemical methods			
Oxidation	Application of strong oxidants (hydrogen peroxide, potassium permanganate ozone gas, or persulfates)	(i) *In situ*/*ex situ* (ii) Fast	(i) Only for organic contaminants (ii) Destruction of the present ecosystems
Hydrolysis	Mostly alkaline hydrolysis	(i) *In situ*/*ex situ* (ii) Fast	(i) Only for organic contaminants (ii) Destruction of the present ecosystems

## Abbreviations

CBA: Chlorobenzoic acid
PCB: Polychlorinated biphenyl.
